# A degree-based block model and a local expansion optimization algorithm for anti-community detection in networks

**DOI:** 10.1371/journal.pone.0195226

**Published:** 2018-04-18

**Authors:** Jiajing Zhu, Yongguo Liu, Changhong Yang, Wen Yang, Zhi Chen, Yun Zhang, Shangming Yang, Xindong Wu

**Affiliations:** 1 Knowledge and Data Engineering Laboratory of Chinese Medicine, School of Information and Software Engineering, University of Electronic Science and Technology of China, Chengdu, Sichuan, China; 2 Sichuan Center for Disease Control and Prevention, Chengdu, Sichuan, China; 3 School of Computing and Informatics, University of Louisiana at Lafayette, Lafayette, Louisiana, United States of America; Universidade Federal do Rio Grande do Sul, BRAZIL

## Abstract

Anti-community detection in networks can discover negative relations among objects. However, a few researches pay attention to detecting anti-community structure and they do not consider the node degree and most of them require high computational cost. Block models are promising methods for exploring modular regularities, but their results are highly dependent on the observed structure. In this paper, we first propose a Degree-based Block Model (DBM) for anti-community structure. DBM takes the node degree into consideration and evolves a new objective function *Q*(*C*) for evaluation. And then, a Local Expansion Optimization Algorithm (LEOA), which preferentially considers the nodes with high degree, is proposed for anti-community detection. LEOA consists of three stages: structural center detection, local anti-community expansion and group membership adjustment. Based on the formulation of DBM, we develop a synthetic benchmark DBM-Net for evaluating comparison algorithms in detecting known anti-community structures. Experiments on DBM-Net with up to 100000 nodes and 17 real-world networks demonstrate the effectiveness and efficiency of LEOA for anti-community detection in networks.

## Introduction

**Fig 1:**
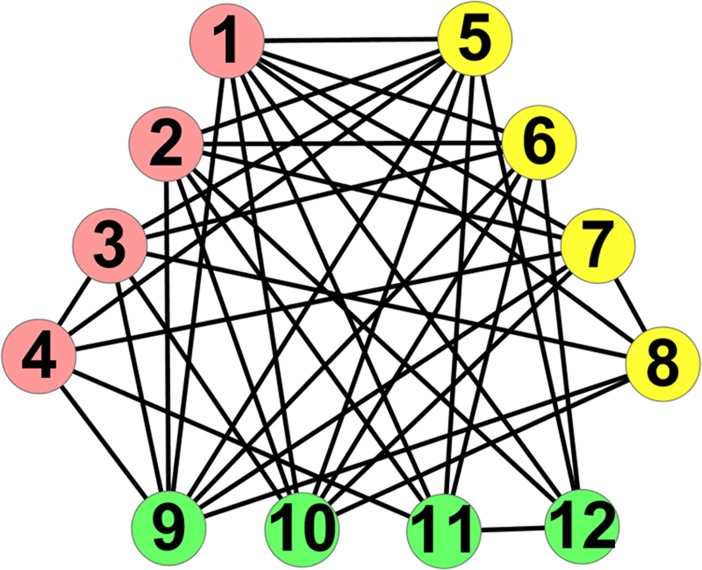
An example of anti-community structure.

The recent researches on complex networks have made significant advancements to our understanding of complex systems [1–3]. Nodes in networks represent the objects, while edges represent the relationships between objects. One of the most important characteristics in complex networks is community structure, i.e. assortative structure [4–6], where nodes share most of their connections inside the groups they belong to. Detecting community structure can reveal the organizational and functional characteristics of underlying systems [7–11]. In this paper, we pay attention to another important structure of complex networks, called anti-community structure, i.e. disassortative structure [12], where nodes have no or few connections with each other inside their group but share most of their connections to the rest of the network as shown in Fig 1. Many real-world networks own the characteristics of anti-community structure [13], such as sexually transmitted disease network, book selling network, and divorce network, etc. Detecting anti-community structure in networks can help reveal some interesting relations, such as non-cooperative relation, competitive relation, and even hostile relation among individuals, corporations, or countries. For example, *Karate* describes the friendship relations between 34 members of a karate club at an American university in the 1970s, which is split into two communities due to the disagreement between the administrator and the instructor [14]. Detecting anti-community structure in *Karate* can divide the members into several groups with no or few friendship relations inside. In each group, some negative relations can be explored among the members, such as the disagreement between the administrator and the instructor.

Several anti-community detection methods have been developed in past few years. These methods attempt to explore anti-community structure in networks from different perspectives. The traditional methods divide a network into two groups to find the largest bipartite structure, which are similar to but not equivalent to the problem of searching for the maximum cut in networks [15–17]. Spectral methods detect anti-community structure by using the negative eigenvalues and eigenvectors of modularity matrix [12, 18]. Label propagation algorithms spread the labels of nodes to the non-neighbor ones to explore multipartite structure in networks [13]. Multipartite structure consists of several groups without internal edge, which is a special case of anti-community structure. Recently, several block models have been proposed for exploring structural regularities in networks [19–26]. These models regard the network structure as observed quantities and take the group membership of nodes as hidden quantities. The structural regularities can be inferred from the group membership. And the group membership of nodes can be inferred by fitting the models to the observed structure based on the method of maximum likelihood such as expectation-maximization (EM) algorithm [27].

However, the above researches suffer from some limitations. First, there is no universally definition for anti-community and no widely-accepted objective function for evaluation. Second, the proposed works [12–13, 15–17, 18–27] do not consider the impacts of node degree on the methods, leading to poor performance especially when they are applied to real-world networks. Thirdly, the efficiency of these methods is comparatively low due to the massive computational cost for calculating of eigenvalues and eigenvectors of modularity matrix in spectral methods and repeated iterations of EM algorithm in block models. In addition, the results provided by block models are highly dependent on the observed structure of a network. For example, block models cannot identify the disassortative structure in *Karate*, because the observed structure in *Karate* is assortative and these methods are incapable of exploring the particular structure that is inconsistent with the observed one. Meanwhile, it is necessary for EM algorithm of block models to run several times with different initial values of parameters to avoid convergence to local optima and find the quantities that fit the observed structure to the most, which also leads to the high computational cost when applied to large networks.

In this paper, we first introduce a definition of anti-community. And then, we propose a Degree-based Block Model (DBM) for anti-community structure, which takes the node degree into consideration and evolves an objective function *Q*(*C*) for anti-community structure evaluation. Due to that the nodes with high degree have greater impacts on *Q*(*C*) than the ones with low degree, a Local Expansion Optimization Algorithm (LEOA), which preferentially considers the nodes with high degree, is proposed for anti-community detection. In LEOA, we first detect structural centers by node influence. Then, LEOA expands each structural center into anti-community by a local search method. Finally, we adjust group membership of nodes by maximizing *Q*(*C*) so as to detect a better anti-community structure. Inspired by the formulation of DBM, a new synthetic benchmark DBM-Net is developed for testing algorithms in detecting known anti-community structure. Experimental results on DBM-Net with up to 100000 nodes and 17 real-world networks demonstrate the effectiveness and efficiency of LEOA for exploring anti-community structure in networks.

The remainder of this paper is organized as follows. We present the related works about anti-community detection in Section 2. Section 3 introduces the definition of anti-community, the formulation of DBM model and the details of LEOA algorithm. The experimental results are described in Section 4. Section 5 gives the conclusions.

## Related works

Some approaches have been proposed for anti-community detection in networks. When a network consists of two anti-communities, the problem is to explore the largest bipartite subgraph in a given network. The detection of bipartite or approximately bipartite structure has attracted attention in the recent literature [15–17]. Searching for the max-cut is an approximate method for solving this problem. Trevisan [15] proposed an approximate algorithm for max-cut by the smallest eigenvalue with approximation ratio of 0.531. Alon and Sudakov [16] obtained two results of dealing with the relation between the smallest eigenvalue of the adjacency matrix of a graph and its bipartite subgraphs. The first result is that the smallest eigenvalue *μ* of the adjacency matrix of any non-bipartite graph with *n* nodes, diameter *L* and its maximum degree *d*_max_ satisfied *μ*≥−*d*_max_+1/((*L*+1)*n*). The other is that they determined the approximation of the max cut algorithm [28] for graph *G* = (*V*,*E*),in which the size of the max-cut is *αm*, where *m* = |*E*|and *α* ∈[0.845,1].Newman [12] used the least negative eigenvalue of modularity matrix for bipartite structure detection in networks. By applying the proposed algorithm to the co-occurrence network of *Nouns and adjectives* in the novel *David Copperfield*, the author found that the obtained partition is approximately bipartite, where one group is almost composed of adjectives and the other of nouns. In addition to the algorithms for bipartite networks, a label propagation algorithm LPAD is proposed by Chen et al. [13] for detecting the partition with more than two anti-communities. LPAD defines the compatible relationship and update rules of labels among nodes, which avoids oscillation in label propagation. The experimental results show that LPAD can detect bipartite and simple multipartite structure in networks but its results are affected by the order of label propagation.

Block models are promising methods for exploring modular regularities in networks [19–26]. However, most of the models focus on the detection of community structure and only two researches can discover disassortative structure [23, 26]. Newman and Leicht [23] proposed a mixture model for exploring broad types of structure in networks. This model takes the assumption that the nodes in the same group have similar connection preference. Due to that this model only considers the relationship between groups and nodes, it may generate the results with mixture of several types of structures, such as assortative structure, disassortative structure, hierarchical structure and core-periphery structure, etc. Shen et al. [26] modified this model and proposed general stochastic block model (GSBM) to detect intrinsic structural regularities of networks. By utilizing the block matrix to indicate the relationship among groups, GSBM can output the types of identified structural regularities.

In this paper, we propose a Local Expansion Optimization Algorithm (LEOA) for anti-community detection in networks by preferentially considering the nodes with high degree, which improves its effectiveness for anti-community detection in synthetic and real-world networks. By first detecting structural centers, then expanding structural centers into anti-communities, and finally adjusting group membership of nodes, LEOA achieves good performance and overcomes the shortcomings of the existing algorithms, such as poor performance in real-world networks, great requirement of computational cost, and high dependency of the observed structure.

## Methods

### Anti-community

Generally, an anti-community can be defined as a group of nodes with most of their connections outside and few or no connections inside. Inspired by the definition of community proposed by Radicchi et al. [29], we provide a quantitative description for anti-community in this subsection.

Consider an undirected and unweighted graph *G* = (*V*,*E*) with *V* being the set of nodes with *n* nodes and *E* = {(*v*_*i*_,*v*_*j*_)|*v*_*i*_,*v*_*j*_∈*V*} being the set of edges with *m* edges, which can be represented as an adjacent matrix **A** such that if there is an edge between node *v*_*i*_ and node *v*_*j*_, *a*_*ij*_ = 1,otherwise *a*_*ij*_ = 1. Let us consider a group *c*_*r*_∈*V*, which *v*_*i*_ belongs to, the degree of node *v*_*i*_ can be written as

$$d_{i}=\sum_{s}m_{i}(s),$$ (1)

where *m*_*i*_(*s*) is the number of edges connecting node *v*_*i*_ to the nodes in group *c*_*s*_

$$m_{i}(s)=\sum_{v_{j}\in c_{s}}a_{ij}.$$ (2)

Thus, group *c*_*r*_ is an anti-community if it satisfies the constraint as follow

$$\lambda \sum_{v_{i}\in c_{r}}m_{i}(r)\leq \underset{s,s\neq r}{\min}\sum_{v_{i}\in c_{r}}m_{i}(s),$$ (3)

where $\sum_{v_{i}\in c_{r}}m_{i}(r)$ is twice the number of edges inside group *c*_*r*_, $\sum_{v_{i}\in c_{r}}m_{i}(s)$ is the number of edges connecting the nodes in group *c*_*r*_ and the nodes in group *c*_*s*_(*s*≠*r*). Eq (3) is regulated by the factor *λ*(*λ* ≥ 1). Given the value of $\underset{s,s\neq r}{\min}\sum_{v_{i}\in c_{r}}m_{i}(s)$, the larger the factor *λ*, the less the number of edges inside group *c*_*r*_, and the better the anti-community *c*_*r*_. And given the value of *λ*, the higher the value of $\underset{s,s\neq r}{\min}\sum_{v_{i}\in c_{r}}m_{i}(s)-\lambda \sum_{v_{i}\in c_{r}}m_{i}(r),$ the better the anti-community *c*_*r*_.

### Degree-based block model

In DBM, given *K* anti-communities, a *K*×*K* matrix **Ω**is adopted and its element *ω*_*rs*_ denotes the probability of edges connecting group *c*_*r*_ and group *c*_*s*_, *r*,*s* = 1,2,…,*K*. Specifically, *ω*_*rr*_ is the probability of edges inside group *c*_*r*_. The probability of an edge connecting node *v*_*i*_ and node *v*_*j*_ is *d*_*i*_*d*_*j*_/(2*m*)^2^ if edges are placed at random. Thus, the probability of an edge connecting node *v*_*i*_ and node *v*_*j*_ with *v*_*i*_∈*c*_*r*_,*v*_*j*_∈*c*_*s*_ is

$$P_{ij}=\omega_{rs}\times \frac{d_{i}d_{j}}{{\left(2m\right)}^{2}},\;v_{i}\in c_{r},v_{j}\in c_{s}.$$ (4)

Since the probability of an edge connecting node *v*_*i*_ and node *v*_*j*_ independently meets a Poisson distribution [22] with the mean of *P*_*ij*_, the possibility of generating graph *G* with edges inside and among anti-communities can be written as follows

$$P(G^{in}|\Omega ,g)=\underset{i\neq j,v_{i},v_{j}\in c_{r}}{\Pi }\frac{{\left(P_{ij}\right)}^{a_{ij}}}{a_{ij}!}\exp(-P_{ij}),$$ (5)

$$P(G^{out}|\Omega ,g)=\underset{\begin{array}{l} i\neq j,r\neq s\\ v_{i}\in c_{r},v_{j}\in c_{s} \end{array}}{\Pi }\frac{{\left(P_{ij}\right)}^{a_{ij}}}{a_{ij}!}\exp(-P_{ij}),$$ (6)

where *a*_*ij*_∈{0,1} and *a*_*ij*_! = 1. Eqs (5) and (6) can be written as follows after manipulations of the equations

$$P(G^{in}|\Omega ,g)=\underset{r}{\Pi }\underset{v_{i}\in c_{r}}{\Pi }{\left(d_{i}\right)}^{2m_{i}(r)}\times \underset{r}{\Pi }[{\left(\omega_{rr}\right)}^{m_{rr}}{\left(\frac{1}{4m^{2}}\right)}^{m_{rr}}\exp(-\omega_{rr}\times \frac{{\left(D_{r}\right)}^{2}-\sum_{v_{i}\in c_{r}}{\left(d_{i}\right)}^{2}}{4m^{2}})],$$ (7)

$$P(G^{out}|\Omega ,g)=\underset{r}{\Pi }\underset{v_{i}\in c_{r}}{\Pi }{\left(d_{i}\right)}^{2m_{i}^{out}(r)}\underset{r,s,r\neq s}{\Pi }[{\left(\omega_{rs}\right)}^{m_{rs}}{\left(\frac{1}{4m^{2}}\right)}^{m_{rs}}\exp(-\omega_{rs}\times \frac{D_{r}D_{s}}{4m^{2}})].$$ (8)

where *m*_*rr*_ is twice the number of edges inside group *c*_*r*_, *m*_*rs*_ is the number of edges between group *c*_*r*_ and group *c*_*s*_, *D*_*r*_ is the group degree of group *c*_*r*_, $m_{i}^{out}(r)$ is the number of edges connecting node *v*_*i*_ to the nodes not belonging to *c*_*r*_. These variables are calculated as follows

$$m_{rr}=\sum_{v_{i}\in c_{r}}m_{i}(r),$$ (9)

$$m_{rs}=\sum_{v_{i}\in c_{r}}m_{i}(s),s\neq r,$$ (10)

$$D_{r}=\sum_{v_{i}\in c_{r}}d_{i},$$ (11)

$$m_{i}^{out}(r)=\sum_{s=1,s\neq r}^{K}m_{i}(s).$$ (12)

Thus, the probability of generating graph *G* parameterized by **Ω** and *g* can be written as follow after multiplying Eqs (7) and (8)

$$P(G|\Omega ,g)=P(G^{in}|\Omega ,g)\times P(G^{out}|\Omega ,g)=\frac{1}{{\left(4m^{2}\right)}^{2m}}\underset{v_{i}}{\Pi }{\left(d_{i}\right)}^{2d_{i}}$$

$$\times \underset{r}{\Pi }[{\left(\omega_{rr}\right)}^{m_{rr}}\exp(-\omega_{rr}\times \frac{{\left(D_{r}\right)}^{2}-\sum_{v_{i}\in c_{r}}{\left(d_{i}\right)}^{2}}{4m^{2}})]\underset{r,s,r\neq s}{\Pi }[{\left(\omega_{rs}\right)}^{m_{rs}}\times \exp(-\omega_{rs}\times \frac{D_{r}D_{s}}{4m^{2}})].$$ (13)

Eq (13) is to be maximized with respect to the matrix **Ω** and group membership *g*. However, likelihood maximization cannot be carried out directly with the likelihood itself, but with its logarithm. Neglecting constants and the terms independent of **Ω** and *g*, we obtain the logarithm of Eq (13) as follow

$$\ln P(G|\Omega ,g)=\sum_{r}\left(m_{rr}\ln\omega_{rr}-\omega_{rr}\times \frac{{\left(D_{r}\right)}^{2}-\sum_{v_{i}\in c_{r}}{\left(d_{i}\right)}^{2}}{4m^{2}}\right)+\sum_{r,s,r\neq s}\left(m_{rs}\ln\omega_{rs}-\omega_{rs}\times \frac{D_{r}D_{s}}{4m^{2}}\right).$$ (14)

Here, we first maximize this expression with respect to the matrix **Ω** By using the method of maximum-likelihood estimate, we take partial derivative of the elements in the matrix **Ω** and obtain the estimation values of *ω*_*rr*_ and *ω*_*rs*_

$${\overset{⌢}{\omega }}_{rr}=\frac{4m^{2}m_{rr}}{{\left(D_{r}\right)}^{2}-\sum_{v_{i}\in c_{r}}{\left(d_{i}\right)}^{2}},\;{\overset{⌢}{\omega }}_{rs}=\frac{4m^{2}m_{rs}}{D_{r}D_{s}}.$$ (15)

By first substituting Eq (15) into Eq (14) and then neglecting the constant 2*m*, we obtain the maximization of Eq (14) with respect to group membership *g*

$$\ln P(G|g)=\sum_{r}M_{rr}+\sum_{r,s,r\neq s}M_{rs}.$$

$$M_{rr}=\left\{ \begin{array}{l} m_{rr}\ln\frac{4m^{2}m_{rr}}{{\left(D_{r}\right)}^{2}-\sum_{v_{i}\in r}{\left(d_{i}\right)}^{2}},\;m_{rr}>0\\ 0,m_{rr}=0 \end{array}\right.,M_{rs}=\left\{ \begin{array}{l} m_{rs}\ln\frac{4m^{2}m_{rs}}{D_{r}D_{s}},m_{rs}>0\\ 0,m_{rs}=0 \end{array}\right..$$ (16)

Given the network partition *C*, we normalize ln*P*(*G*|*g*) by dividing it by a constant, twice the number of edges 2*m*, to constrain the value of ln*P*(*G*|*g*) within relatively tight bounds. The normalized objective function can be written as follow

$$Q(C)=\frac{1}{2m}\ln P(G|g).$$ (17)

**Fig 2:**
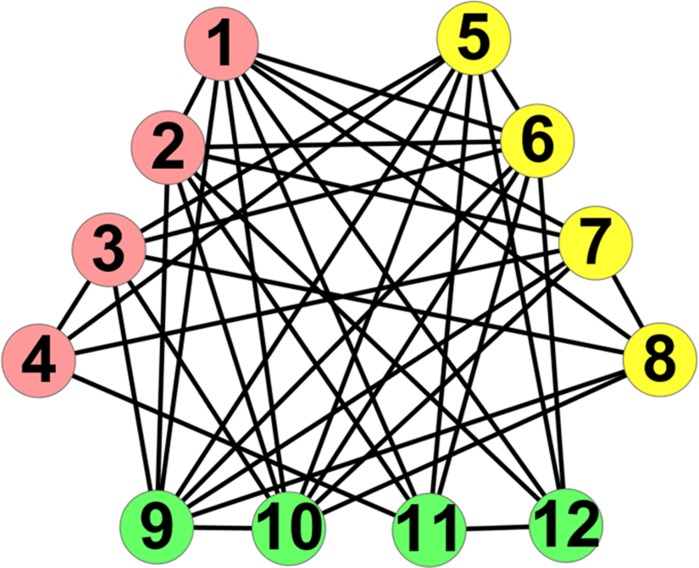
An example of anti-community structure.

**Fig 3:**
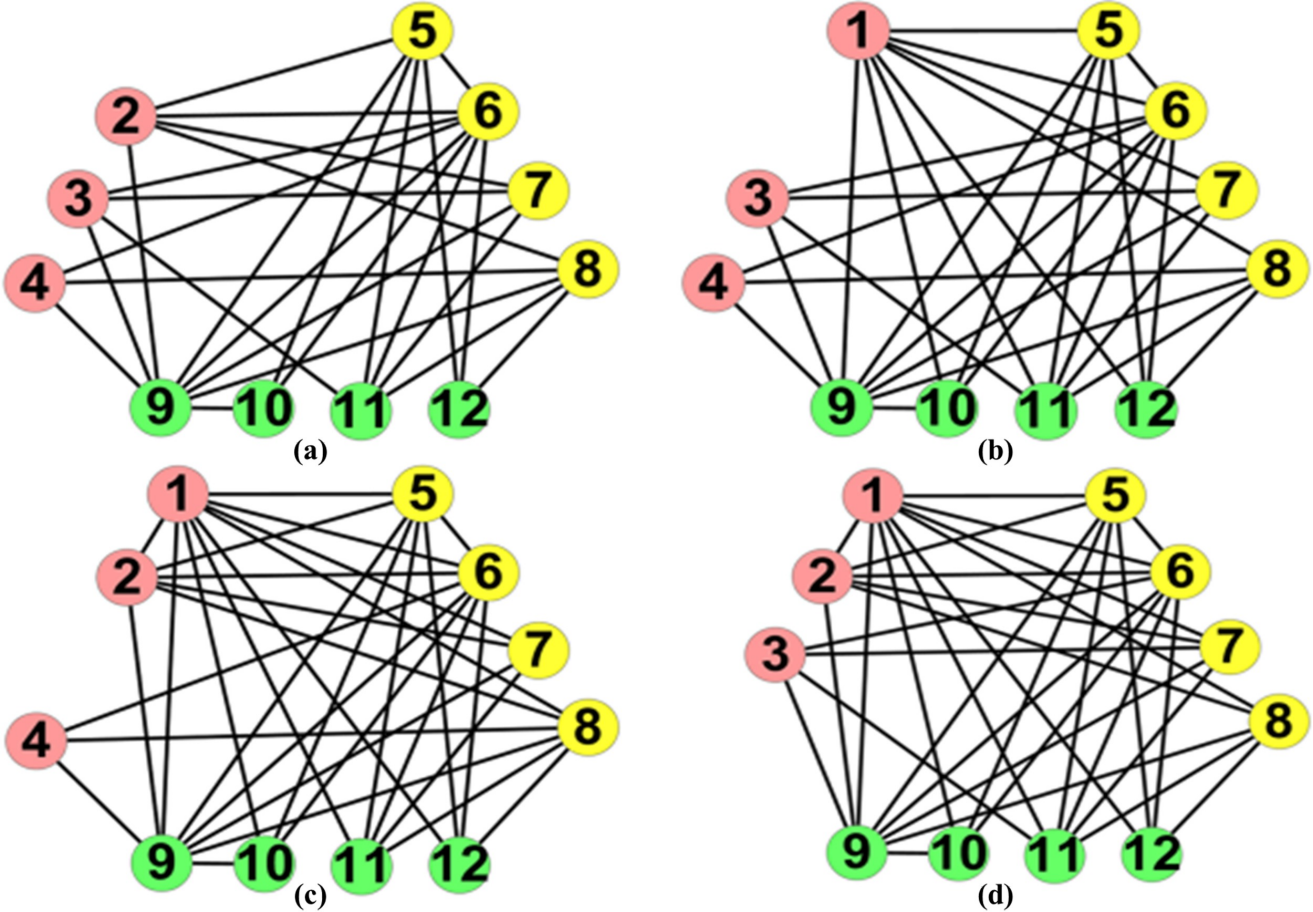
Four anti-community structures. The degree of the four removed nodes *v*_1_, *v*_2_, *v*_3_, *v*_4_ and the values of *Q*(*C*) for the remaining networks are shown in (a), (b), (c), (d) respectively.**(a)** *d*_1_ = 8, *Q*(*C*) = 4.324. **(b**) *d*_2_ = 7, *Q*(*C*) = 4.357. **(c)** *d*_3_ = 6, *Q*(*C*) = 4.448.**(d)** *d*_4_ = 5, *Q*(*C*) = 4.480.

Eq (17) can be considered as a new objective function for evaluating anti-community structure. In Figs 1 and 2, two anti-community structures own the same number of edges and different number of edges inside and among anti-communities. The number of internal edges for each anti-community and the values of *Q*(*C*) for Figs 1 and 2 are shown in Table 1. We observe that the partition in Fig 1 owns the less number of internal edges and a higher value of *Q*(*C*), which indicates that the higher the value of *Q*(*C*), the less the number of internal edges, and the better the anti-community structure. In addition, we find that the nodes with different degree have different impacts on *Q*(*C*). Here, we respectively remove nodes *v*_1_, *v*_2_, *v*_3_ and *v*_4_ from Fig 1 and calculate the values of *Q*(*C*) for the remaining networks as shown in Fig 3. It can be seen that the higher the degree of the removed node, the lower the value of *Q*(*C*) in the remaining network, which indicates that the nodes with high degree have greater contribution to *Q*(*C*) than the ones with low degree. In the proposed algorithm LEOA, we preferentially consider the nodes with high degree so as to be effective for anti-community detection in networks.

**Table 1:** The number of internal edges and the values of *Q*(*C*) for Figs 1 and 2.

Cases	Fig 1			Fig 2		
**Anti-community**	Red	Yellow	Green	Red	Yellow	Green
**Internal Edge**	1	1	1	2	2	2
***Q*(*C*)**	4.567			4.487		

### Local expansion optimization algorithm

**Fig 4:**
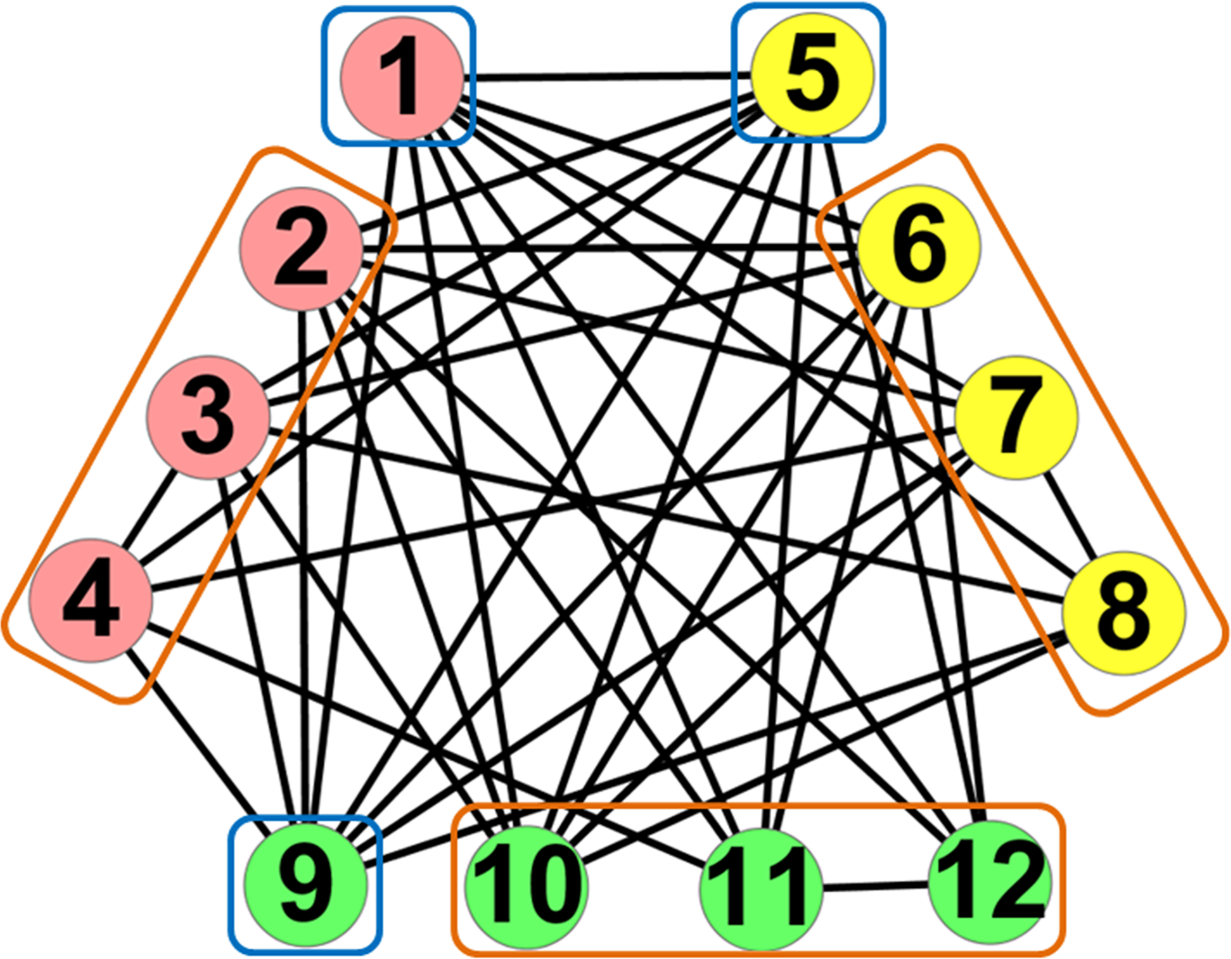
Structural centers and periphery nodes. The nodes in blue boxes are structural centers and the nodes in orange boxes are periphery nodes.

In this paper, we decompose an anti-community into two parts: a central node and several periphery nodes. As shown in Fig 4, node *v*_1_,node *v*_5_ and node *v*_9_ are the central nodes of red, yellow and green anti-communities, respectively, which have no connection to their periphery nodes and are highly connected with each other. Here, we call these central nodes as structural centers. Detecting structural centers plays an important role in anti-community detection. Once structural centers are detected, the number of anti-communities can be determined.

In this subsection, we propose a Local Expansion Optimization Algorithm (LEOA) for detecting anti-community structure in networks. In LEOA, we first detect structural centers by the node influence, which is controlled by a cutoff distance *l*_*c*_. And then, we employ a local search method to detect periphery nodes to expand structural centers into anti-communities. Finally, we adjust the group membership of nodes by maximizing *Q*(*C*) so as to detect a better anti-community structure. The main steps of the proposed algorithm LEOA are given in Algorithm 1.

**Algorithm 1. *Local Expansion Optimization Algorithm* (LEOA).**

**Input:** (*G*,**A**,*l*_*c*_) /* **A** is the adjacent matrix of graph *G* = (*V*,*E*),and *l*_*c*_ is a cutoff distance. */

**Output:***C* = {*c*_1_,*c*_2_,…,*c*_*K*_} /* *C* is the final anti-community structure. */

**1:** (*S*,*K*) = *Structural Center Detection*(*G*,**A**,*l*_*c*_)./* *S* is the set of structural centers and *K* is the number of structural centers.*/

**2:** C* = *Local Anti-community Expansion*(**A**,*l*_*c*_,*S*,*K*).

**3:** *C* = *Group Membership Adjustment*(C*).

**4: return** *C*.

#### Structural Center Detection (SCD)

**Definition 1.** (Node Influence) Consider a graph *G* = (*V*,*E*), the influence *η*_*i*_ of node *v*_*i*_ is a set of nodes within the distance *l*_*c*_ to node *v*_*i*_, which is defined as follow

$$\eta_{i}=\{v_{j}|\delta (l_{c}-l_{ij})=1\},$$ (18)

where *δ*(*x*) = 1 if *x*≥0, and *δ*(*x*) = 0 otherwise. *l*_*c*_ is a cutoff distance, and *l*_*ij*_ denotes the distance between node *v*_*i*_ and node *v*_*j*_. If *l*_*ij*_≤*l*_*c*_, node *v*_*j*_ is influenced by node *v*_*i*_. |*η*_*i*_| is the number of nodes influenced by node *v*_*i*_. The higher the value of *l*_*c*_, the more the number of nodes influenced by node *v*_*i*_, and the higher the value of |*η*_*i*_|. When *l*_*c*_ = *l*,only adjacent nodes of node *v*_*i*_ are influenced by node *v*_*i*_ and |*η*_*i*_| = *d*_*i*_. When *l*_*c*_ = *L*, where *L* is the diameter of the network, |*η*_*i*_| = *n*.

In SCD, structural centers are a set of nodes that influence each other, i.e., the distance among structural centers is no more than *l*_*c*_ When *l*_*c*_ = *l*, structural centers are highly connected with each other and constitute a complete subgraph. Here, we propose an iterative method for structural centers detection. Given the set of structural centers *S*, we define a set of candidate structural centers *CSC* to record the nodes that are influenced by *S*, *CSC* = {*v*_*j*_|*l*_*j*,*S*_≤*l*_*c*_}, where $l_{j,S}=\underset{v_{i}\in S}{\min}(l_{ij}).$ In SCD, the node *v*_*j*_ with $j=\underset{v_{j}\in CSC}{\arg\;\max}(|\eta_{j}|)$ is repeatedly added into *S* until *CSC* = ∅. The main steps of structural centers detection are provided in Algorithm 2. At the beginning, *S* = ∅, *CSC* = ∅ and *K* = 0. *K* is the number of structural centers. First, we calculate the influence of nodes by the breadth-first search method. And then, the node *v*_*i*_ with $i=\underset{v_{i}\in V}{\arg\;\max}(|\eta_{i}|)$ is selected as the first structural center and added to *S*. And we set *CSC*= *η*_*i*_. Next, the node *v*_*j*_ with $j=\underset{v_{j}\in CSC}{\arg\;\max}(|\eta_{j}|)$ is chosen as the second structural center and added into *S*. And we remove node *v*_*j*_ from *CSC*. Since some nodes in *CSC* may not be influenced by node *v*_*j*_, the nodes satisfying {*v*_*k*_|*v*_*k*_∈*CSC*,*l*_*jk*_>*l*_*c*_} are deleted from *CSC* so as to maintain that the nodes in *CSC* are influenced by *S*. We repeatedly execute this operation until *CSC* = ∅ and all structural centers are detected.

**Algorithm 2. *Structural Center Detection* (SCD).**

**Input:**(*G*,**A**,*l*_*c*_) /* **A** is the adjacent matrix of graph *G* = (*V*,*E*), and *l*_*c*_ is a cutoff distance. */

**Output:**(*S*,*K*)/* *S* is the set of structural centers and *K* is the number of structural centers. */

**1:** *S* = ∅,*CSC* = ∅,*K* = 0./* *CSC* is the set of candidate structural centers. */

**2:** Calculate the influence of nodes by the breadth-first search method.

**3:** $i=\underset{v_{i}\in V}{\arg\;\max}(|\eta_{i}|),$ *S* = {*v*_*i*_}, *K* = *K*+1, and *CSC* = *η*_*i*_.

**4: while** *CSC* ≠ ∅ **do**

**5:**     $j=\underset{v_{j}\in CSC}{\arg\;\max}(|\eta_{j}|),$ *CSC* = *CSC*−{*v*_*j*_}.

**6:**     *S* = *S*+{*v*_*j*_}, *K* = *K*+1.

**7:     for** each node *v*_*k*_∈*CSC* **do**

**8:         if** (*l*_*jk*_>*l*_*c*_) **then**

**9:**             *CSC* = *CSC*−{*v*_*k*_}.

**10:         end if**

**11:     end for**

**12:end while**

**13:return** (*S*,*K*).

Here, we take Fig 4 with cutoff distance *l*_*c*_ = 1 as an example to present the procedure of structural centers detection, as shown in Table 2. Initially, *S* = ∅ and *CSC* = ∅. First, we calculate the influence of nodes and find that nodes *v*_1_, *v*_5_ and *v*_9_ own the maximal influence in Fig 4. Then, we randomly select node *v*_1_ as the first structural center and add it to *S*. And the nodes that are influenced by node *v*_1_ are regarded as candidate structural centers and added to *CSC*. In *CSC*, nodes *v*_5_ and *v*_9_ have the maximal influence and we randomly select node *v*_5_ as the second structural center. Thus, we add node *v*_5_ to *S* and remove it from *CSC*. It can be found that nodes *v*_6_, *v*_7_ and *v*_8_ are not influenced by node *v*_5_ due to that the distances between node *v*_5_ and nodes *v*_6_, *v*_7_ and *v*_8_ are more than *l*_*c*_. Therefore, we delete them from *CSC* so as to maintain that the nodes in *CSC* are influenced by *S*. Next, node *v*_9_ has the maximal influence in *CSC* and we select node *v*_9_ as the third structural center and remove it from *CSC*. Due to that distances between node *v*_9_ and nodes *v*_10,_ *v*_11_ and *v*_12_ are more than *l*_*c*_, we delete nodes *v*_10_, *v*_11_ and *v*_12_ from *CSC*. Finally, *CSC* = ∅ and nodes *v*_1_, *v*_5_ and *v*_9_ are detected as structural centers in the network.

**Table 2:** The procedure of structural centers detection in Fig 4.

*S*	∅	{*v*_1_}	{*v*_1_,*v*_5_}	{*v*_1_,*v*_5_}	{*v*_1_,*v*_5_,*v*_9_}	{*v*_1_,*v*_5_,*v*_9_}
*CSC*	∅	{*v*_5_,*v*_6_,*v*_7_,*v*_8_,*v*_9_,*v*_10_,*v*_11_,*v*_12_}	{*v*_6_,*v*_7_,*v*_8_,*v*_9_,*v*_10_,*v*_11_,*v*_12_}	{*v*_9_,*v*_10_,*v*_11_,*v*_12_}	{*v*_10_,*v*_11_,*v*_12_}	∅

#### Local Anti-community Expansion (LAE)

In SCD, *K* structural centers have been detected for *K* anti-communities. In this subsection, we aim to expand the structural centers into anti-communities by a local search method. Here, we define a local anti-community measure, i.e. disassortative density, for local anti-community expansion.

**Definition 2.** (Disassortative Density) For group *c*_*r*_ with *n*_*r*_ nodes and *m*_*r*_edges inside, the disassortative density is defined as follow

$$B_{r}=\frac{\sum_{v_{i}\in c_{r}}\left|\eta_{i}\right|}{2m_{r}+1}.$$ (19)

If *l*_*c*_ = 1, $B_{r}=(\sum_{v_{i}\in c_{r}}d_{i})/(2m_{r}+1).$ Given the value of $\sum_{v_{i}\in c_{r}}\left|\eta_{i}\right|$, the higher the value of *B*_*r*_, the less the number of edges inside group *c*_*r*_, and the more disassortative the group *c*_*r*_.

In LAE, we preferentially consider the nodes with high degree. For each unassigned node *v*_*j*_, we first calculate the increment of disassortative density $\Delta B_{c_{r}+\{v_{j}\}}$ when node *v*_*j*_ is added into group *c*_*r*_, *r* = 1,2,…,*K*. And then we add node *v*_*j*_ into the group *c*_*r*_ with $r=\underset{r=1,2,\ldots ,K}{\arg\;\max}(\Delta B_{c_{r}+\{v_{j}\}})$. If different groups have the same maximal increment of disassortative density, we break this ties by favoring the influence of the group $\sum_{v_{i}\in c_{r}}\left|\eta_{i}\right|$. The increment of disassortative density $\Delta B_{c_{r}+\{v_{j}\}}$ can be calculated in Eq (20) and the main steps of LAE are given in Algorithm 3.

$$\Delta B_{c_{r}+\{v_{j}\}}=\frac{|\eta_{j}|+\sum_{v_{i}\in c_{r}}\left|\eta_{i}\right|}{2m_{j}(r)+2m_{r}+1}-\frac{\sum_{v_{i}\in c_{r}}\left|\eta_{i}\right|}{2m_{r}+1},$$ (20)

where *m*_*j*_(*r*) is the number of edges connecting node *v*_*j*_ and the nodes in group *c*_*r*_.

**Algorithm 3. *Local Anti-community Expansion* (LAE).**

**Input:** (**A**,*l*_*c*_,*S*,*K*)

**Output:** C* = {*c*_1_,*c*_2_,…,*c*_*K*_} /*C* is the anti-community structure after local anti-community expansion. */

**1:** C* = ∅ and *r* = 1.

**2: for** each node *v*_*i*_∈*S* **do** /* Assign *K* structural centers into *K* anti-communities. */

**3:**     *c*_*r*_ = {*v*_*i*_}.

**4:**     C* = C*∪{*c*_*r*_}.

**5:**     *r* = *r*+1.

**6: end for**

**7**: Sort the unassigned nodes in a descending order by the node degree, denoted as *V*.

**8: for** each node *v*_*j*_∈*V* **do**

**9:**     Calculate $\Delta B_{c_{r}+\{v_{j}\}},$ *r* = 1,2,…,*K*.

**10:**     $R=\{r|\underset{r=1,2,\ldots ,K}{\arg\;\max}(\Delta B_{c_{r}+\{v_{j}\}})\},$ $r=\underset{r\in R}{\arg\;\max}(\sum_{v_{i}\in c_{r}}\left|\eta_{i}\right|).$

**11:**     *c*_*r*_ = *c*_*r*_+{*v*_*j*_}.

**12:end for**

**13:return** C*.

#### Group Membership Adjustment (GMA)

As mentioned above, the higher the objective function *Q*(*C*), the better the anti-community structure. In GMA, we aim to adjust the group membership of nodes by maximizing *Q*(*C*) so as to explore a better anti-community structure.

For node *v*_*i*_, we calculate the increment of *Q*(*C*) when node *v*_*i*_ is removed from the group *c*_*r*_ it belongs to and added into a new group *c*_*s*_. The increment value can be calculated as follows

$$\Delta Q{\left(C\right)}_{c_{r}-\{v_{i}\}}^{c_{s}+\{v_{i}\}}=\frac{1}{2m}\Delta \ln P{\left(G|g\right)}_{c_{r}-\{v_{i}\}}^{c_{s}+\{v_{i}\}},$$

$$\Delta \ln P{\left(G|g\right)}_{c_{r}-\{v_{i}\}}^{c_{s}+\{v_{i}\}}=m_{\bar{r}\bar{r}}\ln\frac{4m^{2}m_{\bar{r}\bar{r}}}{{\left(D_{\bar{r}}\right)}^{2}-\sum_{v_{j}\in c_{\bar{r}}}{\left(d_{j}\right)}^{2}}+m_{\bar{s}\bar{s}}\ln\frac{4m^{2}m_{\bar{s}\bar{s}}}{{\left(D_{\bar{s}}\right)}^{2}-\sum_{v_{j}\in c_{\bar{s}}}{\left(d_{j}\right)}^{2}}$$

$$+2m_{\bar{r}\bar{s}}\ln\frac{4m^{2}m_{\bar{r}\bar{s}}}{D_{\bar{r}}D_{\bar{s}}}+2\sum_{k=1,k\neq r,s}^{K}m_{\bar{r}k}\ln\frac{4m^{2}m_{\bar{r}k}}{D_{\bar{r}}D_{k}}+2\sum_{k=1,k\neq r,s}^{K}m_{\bar{s}k}\ln\frac{4m^{2}m_{\bar{s}k}}{D_{\bar{s}}D_{k}}$$

$$-m_{rr}\ln\frac{4m^{2}m_{rr}}{{\left(D_{r}\right)}^{2}-\sum_{v_{j}\in c_{r}}{\left(d_{j}\right)}^{2}}-m_{ss}\ln\frac{4m^{2}m_{ss}}{{\left(D_{s}\right)}^{2}-\sum_{v_{j}\in c_{s}}{\left(d_{j}\right)}^{2}}-2m_{rs}\ln\frac{4m^{2}m_{rs}}{D_{r}D_{s}}$$

$$-2\sum_{k=1,k\neq r,s}^{K}m_{rk}\ln\frac{4m^{2}m_{rk}}{D_{r}D_{k}}-2\sum_{k=1,k\neq r,s}^{K}m_{sk}\ln\frac{4m^{2}m_{sk}}{D_{s}D_{k}},$$ (21)

where $c_{\bar{r}}=c_{r}-\{v_{i}\},$ $c_{\bar{s}}=c_{s}+\{v_{i}\},$ $m_{\bar{r}\bar{r}}$ and $m_{\bar{s}\bar{s}}$ are twice the number of edges inside group $c_{\bar{r}}$ and group $c_{\bar{s}},$ respectively, $D_{\bar{r}}$ and $D_{\bar{s}}$ are group degree of group $c_{\bar{r}}$ and group $c_{\bar{s}},$ respectively, $m_{\bar{r}\bar{s}}$ is the number of edges between group $c_{\bar{r}}$ and group $c_{\bar{s}},$ $m_{\bar{r}k}$ is the number of edges between group $c_{\bar{s}}$ and group *c*_*k*_, $m_{\bar{s}k}$ is the number of edges between group $c_{\bar{s}}$ and group *c*_*k*_. These variables can be computed as follows

$$\left\{ \begin{array}{l} m_{\bar{r}\bar{r}}=m_{rr}-2m_{i}(r)\\ m_{\bar{s}\bar{s}}=m_{ss}+2m_{i}(s)\\ D_{\bar{r}}=D_{r}-d_{i}\\ D_{\bar{s}}=D_{s}+d_{i}\\ m_{\bar{r}\bar{s}}=m_{rs}-m_{i}(s)+m_{i}(r)\\ m_{\bar{r}k}=m_{rk}-m_{i}(k)\\ m_{\bar{s}k}=m_{sk}+m_{i}(k)\\ k=1,2,\ldots ,K,k\neq r,s,r\neq s \end{array}\right.,$$ (22)

where *m*_*i*_(*r*) is the number of edges connecting node *v*_*i*_ and the nodes in group *c*_*r*_, *m*_*i*_(*s*) is the number of edges connecting node *v*_*i*_ and the nodes in group *c*_*s*_, and *m*_*i*_(*k*) is the number of edges connecting node *v*_*i*_ and the nodes in group *c*_*k*_.

For the convenience of calculating $\Delta Q{\left(C\right)}_{c_{r}-\{v_{i}\}}^{c_{s}+\{v_{i}\}}$ in the latter group membership adjustment, we need to update the values of $m_{\bar{r}\bar{r}},$ $m_{\bar{s}\bar{s}},$ $D_{\bar{r}},$ $D_{\bar{s}},$ $m_{\bar{r}\bar{s}},$ $m_{\bar{r}k},$ $m_{\bar{s}k},$ $m_{j}(\bar{r})$ and $m_{j}(\bar{s})$ (*k* = 1,2,…*K*,*k* ≠ *r*,*s* and *a*_*ij*_ = 1), when node *v*_*i*_ is moved from group *c*_*r*_ to group *c*_*s*_.The first seven variables can be updated by Eq (22). $m_{j}(\bar{r})$ and $m_{j}(\bar{s})$ are updated as follows

$$\left\{ \begin{array}{l} m_{j}(\bar{r})=m_{j}(r)-a_{ij}\\ m_{j}(\bar{s})=m_{j}(s)+a_{ij} \end{array}\right..$$ (23)

Due to that the nodes with high degree have greater impacts on *Q*(*C*) than the ones with low degree, the nodes with high degree are preferentially considered here. For each node *v*_*i*_, we calculate $\Delta Q{\left(C\right)}_{c_{r}-\{v_{i}\}}^{c_{s}+\{v_{i}\}}$ (*s* = 1,2,…*K*, and *s* ≠ *r*) and then move node *v*_*i*_ to group *c*_*s*_ with $s=\underset{s=1,2,\ldots ,K,s\neq r}{\arg\;\max(}\Delta Q{\left(C\right)}_{c_{r}-\{v_{i}\}}^{c_{s}+\{v_{i}\}})$ and $\Delta Q{\left(C\right)}_{c_{r}-\{v_{i}\}}^{c_{s}+\{v_{i}\}}>0$. This operation is repeated until no increment of $\Delta Q{\left(C\right)}_{c_{r}-\{v_{i}\}}^{c_{s}+\{v_{i}\}}$ can be found. The main steps of GMA are provided in Algorithm 4.

**Algorithm 4. *Group Membership Adjustment* (GMA).**

**Input:** C*

**Output:** *C* = {*c*_1_,*c*_2_,…,*c*_*K*_}/* *C* is the final anti-community structure. */

**1:** Initialize *m*_*rr*_, *m*_*rs*_ and *m*_*i*_(*r*), *r*,*s* = 1,2,…,*K*,*r* ≠ *s*, and *i* = 1,2,…,*n*.

**2:** Sort nodes in a descending order by the node degree, denoted as *V*, and *C* = C*.

**3: repeat**

**4:**     Δ = 0. /* Δ is used for calculating the sum of $\Delta Q{\left(C\right)}_{c_{r}-\{v_{i}\}}^{c_{s}+\{v_{i}\}}$ for each iteration. */

**5:     for** each node *v*_*i*_∈*V* **do**

**6:**         Calculate $\Delta Q{\left(C\right)}_{c_{r}-\{v_{i}\}}^{c_{s}+\{v_{i}\}},$ *s* = 1,2,…,*K*, and s ≠ *r*./* *c*_*r*_ is the anti-community which node *v*_*i*_ belongs to. */

**7:**         $s=\underset{s=1,2,\ldots ,K,s\neq r}{\arg\;\max}(\Delta Q{\left(C\right)}_{c_{r}-\{v_{i}\}}^{c_{s}+\{v_{i}\}}).$

**8:         if $\left(\Delta Q{\left(C\right)}_{c_{r}-\{v_{i}\}}^{c_{s}+\{v_{i}\}}>0\right)$ then** /* Move node *v*_*i*_ from group *c*_*r*_ to group *c*_*s*._*/

**9:**           *c*_*r*_ = *c*_*r*_−{*v*_*i*_},*c*_*s*_ = *c*_*s*_−{*v*_*i*_}.

**10:**           Update the variables by Eqs (22) and (23).

**11:**           $\Delta =\Delta +\Delta Q{\left(C\right)}_{c_{r}-\{v_{i}\}}^{c_{s}+\{v_{i}\}}.$

**12:         end if**

**13:     end for**

**14: until** Δ = 0.

**15: return** *C*.

### Complexity analysis

In this subsection, we analyze the computational complexity of the proposed algorithm LEOA. Given graph *G* = (*V*,*E*) with *n* nodes and *m* edges, the complexity of calculating the influence of node *v*_*i*_ is $O({\bar{d}}^{l_{c}}),$ where $\bar{d}$ is the average degree of nodes. Thus, it needs $O({\bar{d}}^{l_{c}}n+n)$ to detect structural centers. In LAE, it needs *O*(*n*log*n*) to sort the unassigned nodes in a descending order by the node degree. And for each unassigned node *v*_*i*_, the complexity of assigning node *v*_*i*_ to the group with the maximal increment of its disassortative density is *O*(*d*_*i*_+*K*), where *d*_*i*_ is the degree of node *v*_*i*_. So the complexity of local anti-community expansion is *O*(*n*log*n*+*m*+*nK*). In GMA, the complexity of calculating $\Delta Q{\left(C\right)}_{c_{r}-\{v_{i}\}}^{c_{s}+\{v_{i}\}}$ is *O*(*d*_*i*_+*K*) and the complexity of updating variables by Eqs (22) and (23) is *O*(*d*_*i*_). Thus, it requires *O*(*mK*+*nK*_2_) to adjust the group membership of nodes. The total complexity of LEOA is $O[n({\bar{d}}^{l_{c}}+\log n+K^{2})+mK].$ In our experiments, we find that LEOA achieves the best performance when *l*_*c*_ = 1, so the time complexity of LEOA is *O*(*n*log*n*+*nK*^2^+*mK*).

## Experiments

In this section, we evaluate the performance of LEOA on synthetic benchmark DBM-Net and 17 real-world networks [30–32]. The experiments on DBM-Net aim to test the ability of LEOA to detect known anti-communities, while the experiments on real-world networks are to access its performance in real applications. Here, we compare LEOA with its variant LEOA* and five state-of-the-art anti-community detection algorithms: Spectral [18], Di-Spectral [12], E-Model [26], M-Model [23] and LPAD [13]. LEOA* does not take the node degree into consideration and randomizes the node order for LAE and GMA. Spectral and Di-Spectral utilize negative eigenvalues and eigenvectors of modularity matrix for anti-community detection. E-Model and M-Model are two block models for structural regularities detection optimized by EM algorithm. LPAD is a recently proposed anti-community detection algorithm based on label propagation. Due to that EM often converges to local optima, we repeatedly carry out EM algorithm 20 times with different initial values for E-Model and M-Model and output the best result for each network. All algorithms are independently run 20 times for each experimental network. The comparison algorithms are conducted by C# on a PC with Intel (R) Core i5-4460 3.20 GHz and 4GB real memory.

As DBM-Net and real-world disassortative networks have known anti-community structures, we adopt the Normalize Mutual Information [33] (NMI) to estimate the similarity between the true partition and the detected one. Assuming that the true partition of a network with *n* nodes is *C*_1_ and the detected one is *C*_2_, *NMI*(*C*_1_,*C*_2_) can be computed as

$$NMI(C_{1},C_{2})=\frac{-2\sum_{i=1}^{K_{C_{1}}}\sum_{j=1}^{K_{C_{2}}}f_{ij}\log[f_{ij}n/(f_{i\cdot }f_{\cdot j})]}{\sum_{i=1}^{K_{C_{1}}}f_{i\cdot }\log(f_{i\cdot }/n)+\sum_{i=1}^{K_{C_{2}}}f_{\cdot j}\log(f_{\cdot j}/n)},$$ (24)

where **F** is a confusing matrix, its element *f*_*ij*_ records the number of the same nodes of the *i*th group of *C*_1_ and the *j*th group of *C*_2_, *f*_*i*·_(*f*_.*j*_) is the sum of the elements of the *i*th row (*j*th column) in **F,** and $K_{C_{1}}(K_{C_{2}})$ represents the number of groups in partition *C*_1_(*C*_2_). The value of NMI is between [0,1] and the larger value of NMI indicates that the detected structure is more accordant with the true one.

### Datasets

#### Synthetic benchmark DBM-Net

To our knowledge, there is no benchmark designed for anti-community detection. Inspired by the formulation of DBM, we develop a new benchmark called DBM-Net for comparison algorithms in detecting known anti-community structures.

Most of complex networks in real-world are scale-free networks [34], where node degree follows a power law distribution. Thus, we set that the node degree for DBM-Net follows a power law distribution with exponent *β* and coefficient *α*, which means that the probability of randomly selecting a node with *d*_*i*_ degree is *P*(*d*_*i*_) = *α*(*d*_*i*_)^−*β*^. Given the value of exponent *β*, the maximal degree *d*_max_ and the minimal degree *d*_min_, the coefficient *α* can be calculated as follow

$$\alpha =\frac{1}{\sum_{d_{i}=d_{\min}}^{d_{\exp}}{\left(d_{i}\right)}^{-\beta }}.$$ (25)

So the number of nodes with *d*_*i*_ degree is *n*(*d*_*i*_) = ⌊*n*×*P*(*d*_*i*_)⌋, *d*_*i*_∈[*d*_min_,*d*_max_], and the number of edges *m* can be calculated as follow

$$m=\left\lfloor 0.5\sum_{d_{i}=d_{\min}}^{d_{\exp}}d_{i}\left\lfloor n\times P(d_{i})\right\rfloor \right\rfloor ,$$ (26)

Given the number of groups *K*, the number of edges inside and among groups *m*_*rr*_, *m*_*rs*_ (*r*,*s* = 1,2,…,*K*, and *r* ≠ *s*) are constrained by Eq (27).

$$\left\{ \begin{array}{l} \sum_{r=1}^{K}m_{rr}+\sum_{r=1}^{K}\sum_{s=1,s\neq r}^{K}m_{rs}=2m\\ \lambda m_{rr}\leq \underset{s,s\neq r}{\min}m_{rs},r=1,2,\ldots ,K \end{array}\right..$$ (27)

For simplicity, we set that the values of *m*_*rr*_ are the same for *r* = 1,2,…,*K*, and the values of *m*_*rs*_ are also the same for *r*,*s* = 1,2,…,*K*, *r* ≠ *s*. Thus, we obtain (*m*_*rr*_)_min_ = 0 and (*m*_*rr*_)_max_ = ⌊2*m*/(*K*+*λK*^2^−*λK*)⌋. Given the degree of each node, the number of nodes *n*_*r*_ in group *c*_*r*_ satisfies the following constraints

$$\left\{ \begin{array}{l} \sum_{r=1}^{K}n_{r}=n\\ \sum_{r=1}^{K}\sum_{v_{i}\in c_{r}}d_{i}=2m \end{array}\right.,$$ (28)

where $\sum_{v_{i}\in c_{r}}d_{i}=D_{r}.$ Here, we take the assumption that the group degree follows a uniform distribution, i.e., the group degree for group *c*_*r*_ is *D*_*r*_ = ⌊2*m*/*K*⌋, *r* = 1,2,…,*K*. The main steps of establishing synthetic benchmark DBM-Net are described in Algorithm 5.

**Algorithm 5. DBM-Net Establishment.**

**Input:** (*n*,*K*,*m*_*rr*_,*β*,*d*_min_,*d*_max_,*λ*)

**Output:** (*C* = {*c*_1_,*c*_2_,…,*c*_*K*_},**A**) /* *C* is the anti-community structure, **A** is the adjacent matrix. */

**1:** Calculate the coefficient *α* according to Eq (25).

**2:** Calculate the values of *n*(*d*_*i*_)and randomly assign *n*(*d*_*i*_) nodes with *d*_*i*_ degree, *d*_*i*_∈[*d*_min_,*d*_max_].

**3:** Calculate the number of edges *m* according to Eq (26).

**4:** Randomly assign *n*_*r*_ nodes into group *c*_*r*_ with the group degree *D*_*r*_ = ⌊2*m*/*K*⌋, *r* = 1,2,…,*K*.

**5:** Calculate the number of edges *m*_*rs*_ between group *c*_*r*_ and group *c*_*s*_, $m_{rs}=\left\lfloor \frac{2m-Km_{rr}}{K(K-1)}\right\rfloor$, *r*,*s* = 1,2,…*K*,*r* ≠ *s*.

**6:** Calculate the estimation values of *ω*_*rr*_ and *ω*_*rs*_ according to Eq (15).

**7: for** *r* = 1 to *K* **do**

**8:     for** each pair of nodes *v*_*i*_,*v*_*j*_∈*c*_*r*_ **do**

**9:**         Calculate the probability of an edge connecting node *v*_*i*_ and node *v*_*j*_, $P_{ij}=\omega_{rr}\times \frac{d_{i}d_{j}}{{\left(2m\right)}^{2}}.$

**10:**         Generate a random number *P*∈[0,1].

**11:         if** (*P*≤*P*_*ij*_) **then**

**12:**             *a*_*ij*_ = 1./* There is an edge connecting node *v*_*i*_ and node *v*_*i*._*/

**13:         else**

**14:**             *a*_*ij*_ = 0. /* There is no edge connecting node *v*_*i*_ and node *v*_*j*_.*/

**15:         end if**

**16:     end for**

**17:end for**

**18:for** *r*, *s* = 1 to *K* **do** /* *r* ≠ s*/

**19:     for** each pair of nodes *v*_*i*_∈*c*_*r*_,*v*_*j*_∈*c*_*s*_ **do**

**20:**         Calculate the probability of an edge connecting node *v*_*i*_ and node *v*_*j*_, $P_{ij}=\omega_{rs}\times \frac{d_{i}d_{j}}{{\left(2m\right)}^{2}}.$

**21:**         Generate a random number *P*∈[0,1].

**22:         if** (*P*≤*P*_*ij*_) **then**

**23:**             *a*_*ij*_ = 1.

**24:         else**

**25:**             *a*_*ij*_ = 0.

**26:         end if**

**27:     end for**

**28:end for**

**29:return** (*C* = {*c*_1_,*c*_2_,…,*c*_*K*_},**A**).

### Real-world networks

In this paper, we adopt 17 real-world networks [30–32] to evaluate the performance of LEOA, which are divided into two categories: disassortative network and assortative network as shown in Tables 3 and 4, respectively. The experiments on disassortative networks aim at validating the effectiveness of LEOA in exploring known partitions in real applications. Due to that the observed structure in an assortative network is a community structure, the experiments on assortative networks are to test whether LEOA is capable of detecting anti-community structure when the detected structure is inconsistent with the observed one. Here, we adopt NMI and *Q*(*C*) for evaluation in disassortative and assortative networks, respectively.

**Table 3:** Disassortative network.

Network	*n*	*m*	*L*	Network	*n*	*m*	*L*
Southern women	32	89	4	Nouns and adjectives	112	425	5
Divorce in US	59	225	4	Interlocks in Scotland	244	358	∞
Cities and services	101	1342	3	Unicode languages	868	1255	∞

**Table 4:** Assortative network.

Network	*n*	*m*	*L*	Network	*N*	*m*	*L*
Karate	34	78	5	Political blogs	1490	19090	8
Dolphin	62	159	8	Netscience	1589	2742	17
US politics books	105	441	7	Human protein	3133	6726	10
Football	115	613	4	Power	4941	6594	46
Elegans	453	2025	7	DBLP cite	12591	49743	10
Air traffic control	1226	2615	17				

In disassortative networks, (1) *Southern women* describes the participation of 18 women in 14 social events in 1930s. (2) *Divorce in US* illustrates the relationship of 9 main causes of the divorce cases in 50 states of USA. (3) *Cities and services* provides the distribution of offices for 46 global advanced producer service firms over 55 cities. (4) *Nouns and adjectives* describes a co-occurrence network of Nouns and adjectives in the novel *David Copperfield*. (5) *Interlocks in Scotland* characterizes the relationship between 108 Scottish firms and 136 multiple directors during 1904–1905. (6) *Unicode languages* illustrates the usage of 254 languages over 614 territories around the world. Due to that *Interlocks in Scotland* contains 15 isolated nodes and *Unicode languages* consists of 5 connecting components, their diameters *L* are ∞.

In assortative networks, (1) *Karate* is a friendship network between 34 members of a karate club at a US university in the 1970s, which is divided into two communities due to the disagreement between the administrator and the instructor. (2) *Dolphin* is a social network of frequent associations among 62 dolphins living in Doubtful Sound, New Zealand and it is divided into two communities according to their age. (3) *US politics books* describes a frequent co-purchasing network of US politics books by the same buyers in Amazon. The books fall into three types: liberal, neutral, and conservative. (4) *Football* is a network of American football games among 115 Division IA teams during regular season in Fall 2000. The teams are divided into 12 conferences and the games are more frequent among the teams in the same conference than the ones in different conferences. (5) *Elegans* describes the relationship between 453 metabolic molecules in a metabolic process. (6) *Air traffic control* is a network of travel routes among 1226 airports and service centers. (7) *Political blogs* describes a hyperlinks network among 1490 weblogs on US politics. (8) *Netscience* is a collaboration network of scientists working on network theory and experiment. (9) *Human protein* illustrates interactions among 4941 proteins of human; (10) *Power* represents the topology of the Western States Power Grid of USA. (11) *DBLP cite* is a network describing the citations among 12591 publications.

### Performance evaluation

The cutoff distance *l*_*c*_ has great impacts on the number of anti-communities *K*, the computational cost and effectiveness of LEOA. As mentioned in complexity analysis of LEOA, the higher the value of *l*_*c*,_ the higher the computational cost of LEOA. As DBM-Net and real-world disassortative networks have known anti-community structures, we analyze the impacts of cutoff distance *l*_*c*_ on NMI and the number of anti-communities *K* in DBM-Net and real-world disassortative networks. Here, four datasets DBM-Net (*n* = 500, *K* = 2, *m*_*rr*_ = 0, *β* = 2, *d*_min_ = 10, *d*_max_ = 50) with *L* = 5, *Southern women*, *Cities and services* and *Unicode languages* are selected for performance evaluation.

**Fig 5:**
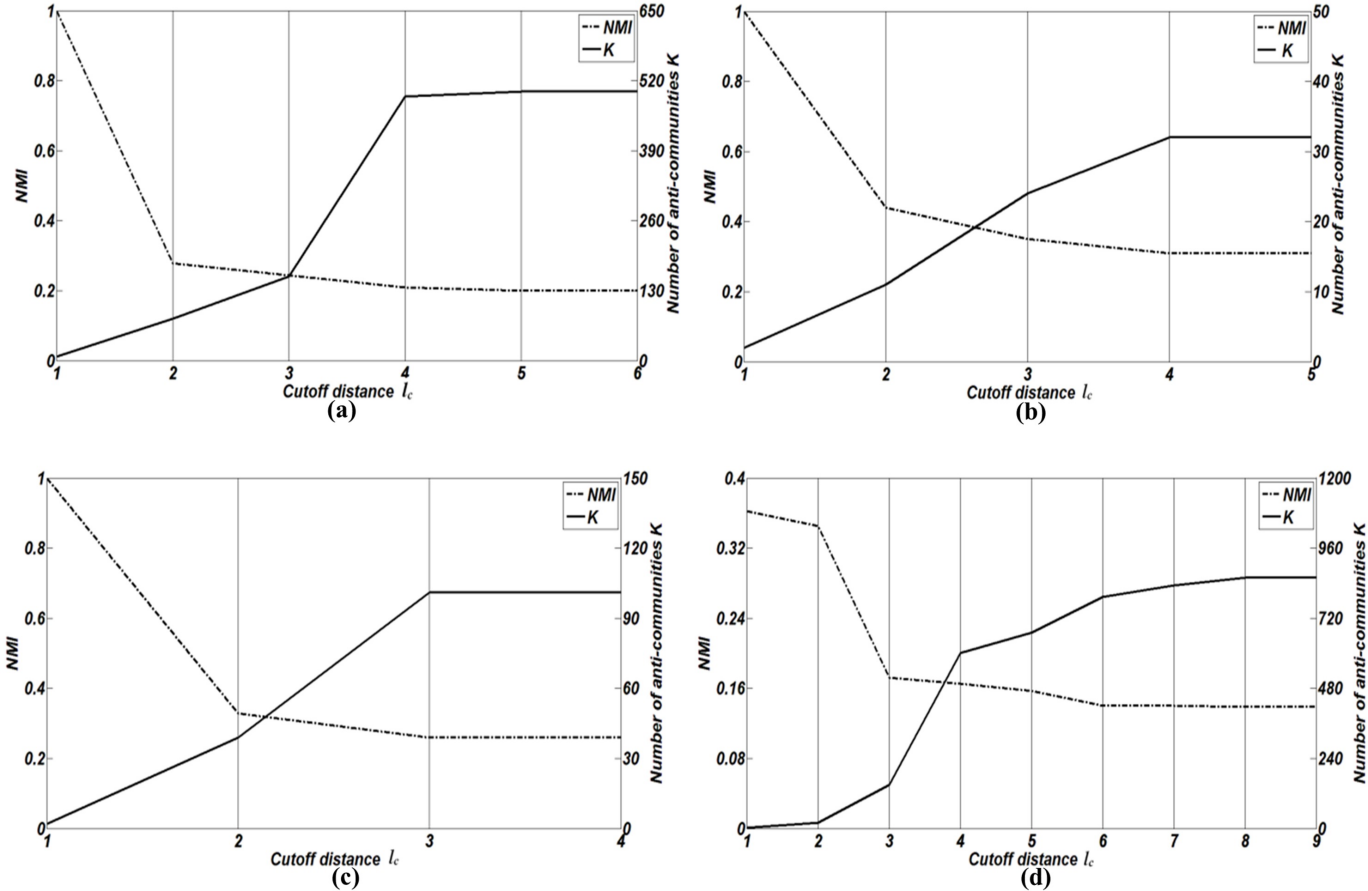
The results of NMI and the number of anti-communities *K* for different values of *l*_*c*_. **(a)** DBM-Net. **(b)** Southern women. **(c)** Cities and services. **(d)** Unicode languages.

Fig 5 shows the results of NMI and *K* for different values of *l*_*c*_ It can be observed that the increase of *l*_*c*_ leads to the decrease of NMI and the increase of *K*. The reason is that as *l*_*c*_ increases, |*η*_*i*_| is also increases, *i* = 1,2,…*n*, leading to the increase of the nodes that influence each other and the increase of the structure centers explored by SCD, which results in the decrease of NMI. When *l*_*c*_ = 1, LEOA outputs two anti-communities in these four networks and the values of NMI are higher than those when *l*_*c*_ = 1. Thus, we set *l*_*c*_ = 1 in this paper. When *l*_*c*_ = *L*, all nodes influence each other and each node forms an anti-community, which leads to the lowest NMI. In addition, we find that the number of nodes that influence each other increases greatly in cases of DBM-Net and *Unicode languages* when 3≤*l*_*c*_≤4. This may explain the results that *K* increases greatly in these two networks when 3≤*l*_*c*_≤4.

### Performance comparison on DBM-Net

**Fig 6:**
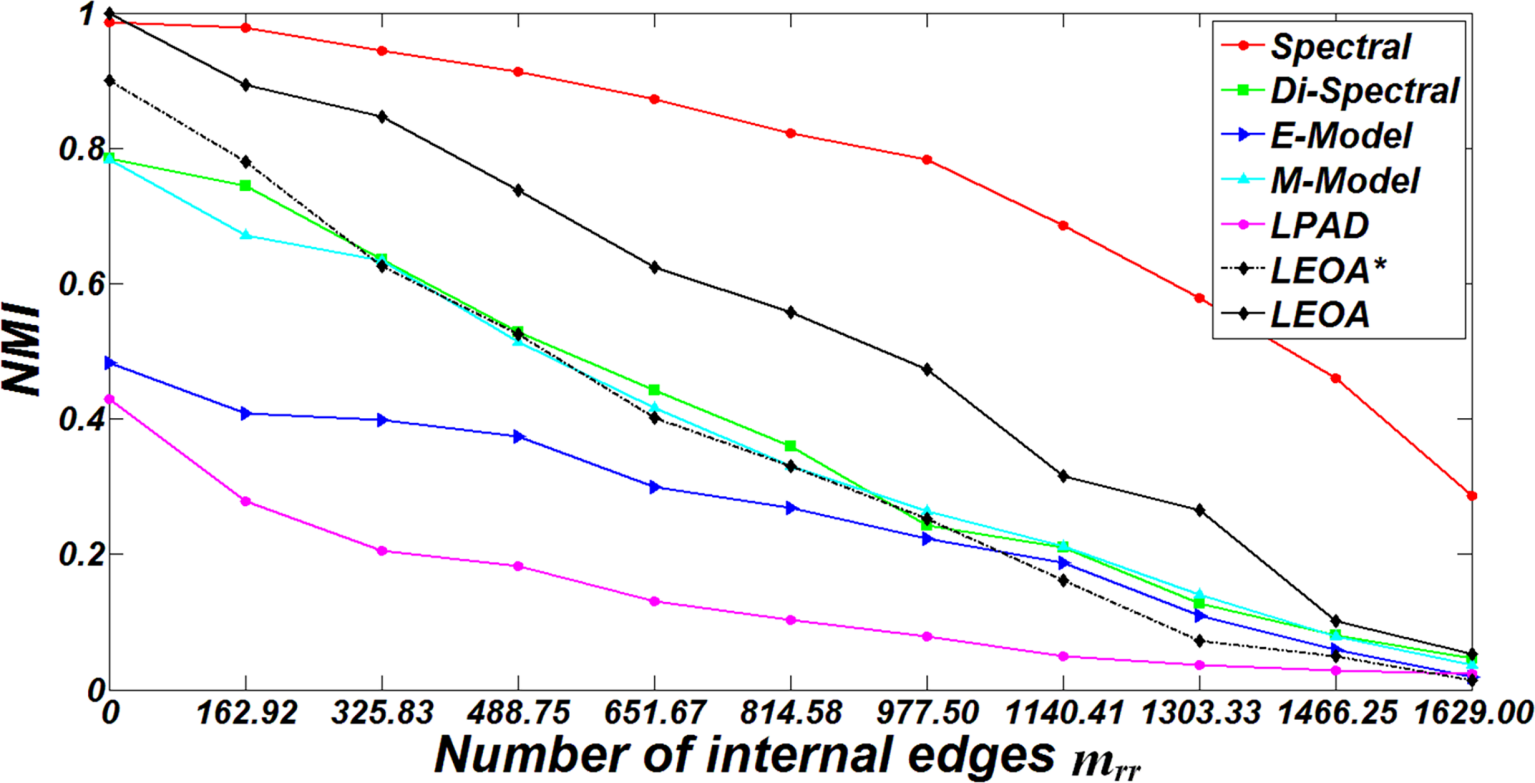
The results of NMI of comparison algorithms on DBM-Net for different values of *m*_*rr*_.

In this subsection, comparison algorithms are applied to DBM-Net to evaluate their performance in detecting known anti-community structure. We first evaluate the performance of comparison algorithms on DBM-Net with the increase of twice the number of internal edges *m*_*rr*_. When *m*_*rr*_ = (*m*_*rr*_)_min_, no edge can be found in each group and DBM-Net degenerates into a multipartite network. When (*m*_*rr*_)_min_<*m*_*rr*_≤(*m*_*rr*_)_max_, *λm*_*rr*_ is less than or equal to *m*_*rs*_ (*s* = 1,2,…,*K*, and *r* ≠ *s*) and DBM-Net is a network with anti-community structure according to Eq (3). When *m*_*rr*_>(*m*_*rr*_)_max_, DBM-Net does not have the characteristics of anti-community structure anymore. For comparison, we set *n* = 500, *K* = 2, *β* = 2, *d*_min_ = 10, *d*_max_ = 50, *λ* = 2 and *m*_*rr*_ varies from (*m*_*rr*_)_min_ to (*m*_*rr*_)_max_ with an increment of (*m*_*rr*_)_max_/10. For each value of *m*_*rr*,_ 20 networks are generated and the results of comparison algorithms are shown in Fig 6. It can be observed that the increase of *m*_*rr*_ leads to the decrease of NMI because internal edges weaken the anti-community structure and increase the difficulty of anti-community detection. It can be seen that Spectral outputs higher values of NMI than LEOA except *m*_*rr*_ = (*m*_*rr*_)_min_. The reason is that when *m*_*rr*_ = (*m*_*rr*_)_min_, the number of structural centers detected by SCD is equal to the number of groups in the true partition, which helps LAE and GMA to find the true partition. When *m*_*rr*_>(*m*_*rr*_)_min_, there are some edges inside each group in the true partition and the number of structural centers detected by SCD may be more than the number of groups in the true partition, which results in that some groups in the true partition may be split into several small groups and the values of NMI decrease. We observe that the higher the value of *m*_*rr*_, the more the number of structural centers detected by SCD, and the lower the value of NMI. Due to that the number of anti-communities explored by Di-Spectral is much more than the one in the true partition, its values of NMI are lower than those output by Spectral and LEOA. Although EM algorithm is repeatedly carried out with different initial values for E-Model and M-Model, it is still easy for them to fall into local optima and the results output by these two algorithms rely on the threshold of EM algorithm. In addition, we find that the values of NMI output by LPAD are lower than those output by other algorithms in most cases. On one hand, LPAD selects compatible nodes for label updation but the order of compatible nodes selection has great impacts on its accuracy. On the other hand, no internal edge is allowed in the results output by LPAD, which leads to that the higher the value of *m*_*rr*_, the more the number of groups detected by LPAD, and the lower the value of NMI. It can be seen that the values of NMI provided by LEOA* are lower than those provided by LEOA, which indicates that consideration of node degree in LAE and GMA can improve the effectiveness of LEOA for anti-community detection in DBM-Net.

**Fig 7:**
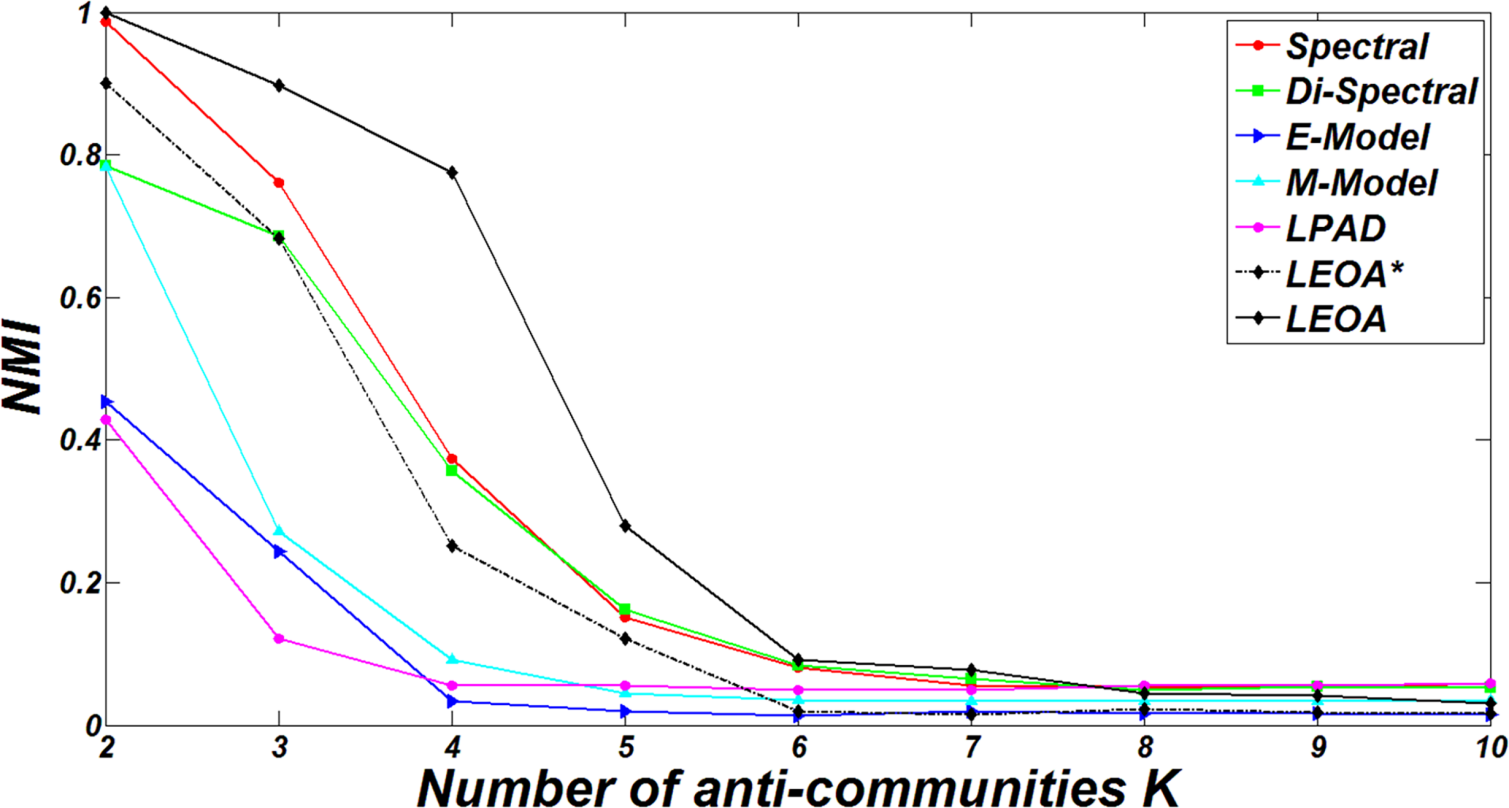
The results of NMI of comparison algorithms on DBM-Net for different values of *K*.

To further verify the effectiveness of LEOA in detecting known anti-community structures, we apply the comparison algorithms to DBM-Net with the increase of the number of groups *K*. When *K* = 1, DBM-Net consists of only one anti-community. And when *K* = *n*, each node forms an anti-community. For comparative experiments, we set *n* = 500, *m*_*rr*_ = 0, *β* =2, *d*_min_ = 10, *d*_max_ = 50, and *K* varies from 2 to 10. The NMI results of comparison algorithms are shown in Fig 7. It can be seen that with the increase of *K*, it becomes more and more difficult for the algorithms to detect the true partition. The reason is that as *K* increases, each node has a higher probability to be assigned to a wrong group, especially in the early stage of the algorithms. And when *K*≥7, all algorithms fail to find the true partition (NMI≈0). It can be observed that when 2≤*K*≤4, the NMI results of LEOA fall more slowly than those of other algorithms, but when 4<*K*≤6, the NMI results of LEOA fall faster than those of other algorithms. The reason is that when 2≤*K*≤4, the number of structural centers detected by SCD is equal to the number of groups in the true partition, leading to high values of NMI (*NMI*≥0.8) and a slow descent of NMI. In cases of 4<*K*≤6, LEOA cannot detect the structural centers for some groups in the true partition, because all nodes in these groups are not highly connected with the structural centers in other groups, which leads to the wrong assignments of the nodes and a fast descent of NMI.

**Fig 8:**
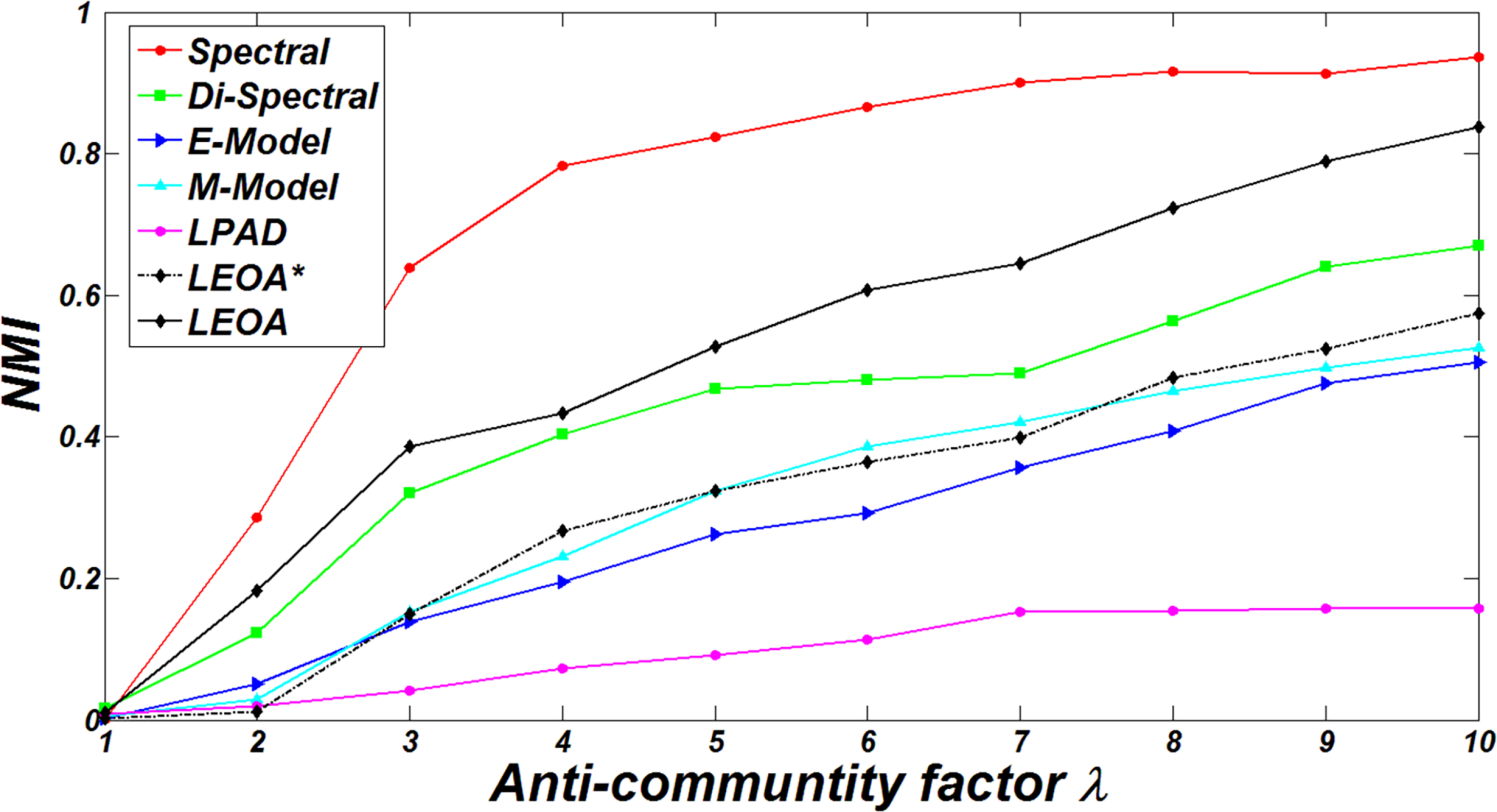
The results of NMI of comparison algorithms on DBM-Net for different values of *λ*.

As mentioned above, the factor *λ* in Eq (3) controls the number of edges inside and among anti-communities. Here, we evaluate the performance of comparison algorithms in DBM-Net with the increase of the factor *λ* For comparison, we set *n* = 500, *K* = 2, *β* =2, *d*_min_ = 10, *d*_max_ = 50, *λm*_*rr*_ = *m*_*rs*_ (*s* = 1,2,…,*K*, and *r* ≠ *s*)and *λ* varies from 1 to 10. The results of NMI of comparison algorithms are shown in Fig 8. It can be observed that the increase of *λ* leads to the increase of NMI. Given the number of edges *m*, the higher the value of *λ*, the fewer the number of edges inside groups, and the more the number of edges among groups, which is easier for the algorithms to detect the true partition and leads to high values of NMI.

### Performance comparison on real-world networks

Table 5 shows the results of comparison algorithms on 6 disassortative networks. It can be observed that all algorithms output the true partitions for the first three networks. In the remaining networks, LEOA provides the highest values of NMI. It can be found that the NMI results of all algorithms on *Nouns and adjectives* are less than 0.4. The reason is that there are some edges among nouns nodes and some edges among adjectives nodes, which leads to an incomplete bipartite network and increases the difficulty of the algorithms to explore the true partition. As LAE and GMA may generate some edges inside groups, which is suitable to *Nouns and adjectives*, LEOA provides a higher NMI than others. We observe that the values of NMI of all algorithms on *Interlocks in Scotland* are less than 0.5. The main reason is that *Interlocks in Scotland* contains 15 isolated nodes, which affect the calculation of eigenvalues and eigenvectors of modularity matrix for Spectral and Di-Spectral and the calculation of maximum likelihood optimized by EM algorithm for E-Model and M-Model. Due to that the isolated nodes are compatible with any other node, LPAD cannot accurately determine the labels for these nodes. In addition, LEOA always assigns the isolated nodes to the group with the maximal group size so as to output higher *Q*(*C*). These reasons result in the wrong assignments of isolated nodes and even affect the assignments of other nodes, leading to the low values of NMI. In addition, we find that all algorithms cannot detect the true partition in *Unicode languages*. The reason is that *Unicode language* consists of 5 connected components with bipartite structure, leading to that 16 different partitions can be obtained by randomly combining the connected components into a final bipartite structure. And the bipartite structures detected by the comparison algorithms are different from the true one. It can be observed that the NMI results provided by LEOA are higher than those provided by LEOA* in the last three networks, which demonstrates that node degree factor in LEOA can enhance the accuracy of LEOA. From these results, we can see that LEOA achieves good performance for anti-community detection in experimental disassortative networks.

**Table 5:** Experimental results of comparison algorithms on disassortative networks.

Datasets	*NMI*						
	Spectral	Di-Spectral	E-Model	M-Model	LPAD	LEOA*	LEOA
Southern women	**1.000**	**1.000**	**1.000**	**1.000**	**1.000**	**1.000**	**1.000**
Divorce in US	**1.000**	**1.000**	**1.000**	**1.000**	**1.000**	**1.000**	**1.000**
Cities and services	**1.000**	**1.000**	**1.000**	**1.000**	**1.000**	**1.000**	**1.000**
Nouns and adjectives	0.191	0.311	0.022	0.203	0.095	0.303	**0.323**
Interlocks in Scotland	0.041	0.051	0.204	0.317	0.106	0.285	**0.455**
Unicode languages	0.163	0.286	0.241	0.297	0.031	0.292	**0.362**

Table 6 shows the results of the comparison algorithms on 11 assortative networks. Due to that the observed structure in an assortative network is a community structures and the results output by E-Model and M-Model are highly dependent on the observed one of a network, they cannot output anti-community structure on an assortative network and their results are not considered here. It can be seen that the values of *Q*(*C*) provided by LEOA are higher than those provided by other algorithms, which indicates that LEOA is superior to other algorithms for experimental assortative networks.

**Table 6:** Experimental results of comparison algorithms on assortative networks.

Datasets	*Q*(*C*)				
	Spectral	Di-Spectral	LPAD	LEOA*	LEOA
Karate	5.249	5.308	5.286	5.337	**5.351**
Dolphin	5.863	5.833	5.962	6.009	**6.165**
US politics books	6.910	6.797	6.971	6.972	**7.000**
Football	7.173	7.130	7.266	7.637	**7.872**
Elegans	8.416	8.371	8.484	8.811	**8.835**
Air traffic control	8.544	8.551	8.770	8.774	**8.778**
Political blogs	10.440	10.484	10.506	11.084	**11.091**
Netscience	8.975	8.611	8.889	9.805	**9.877**
Human protein	9.499	9.645	9.586	9.664	**9.687**
Power	9.657	9.781	9.880	9.878	**9.886**
DBLP cite	8.674	8.762	8.772	8.773	**8.779**

**Fig 9:**
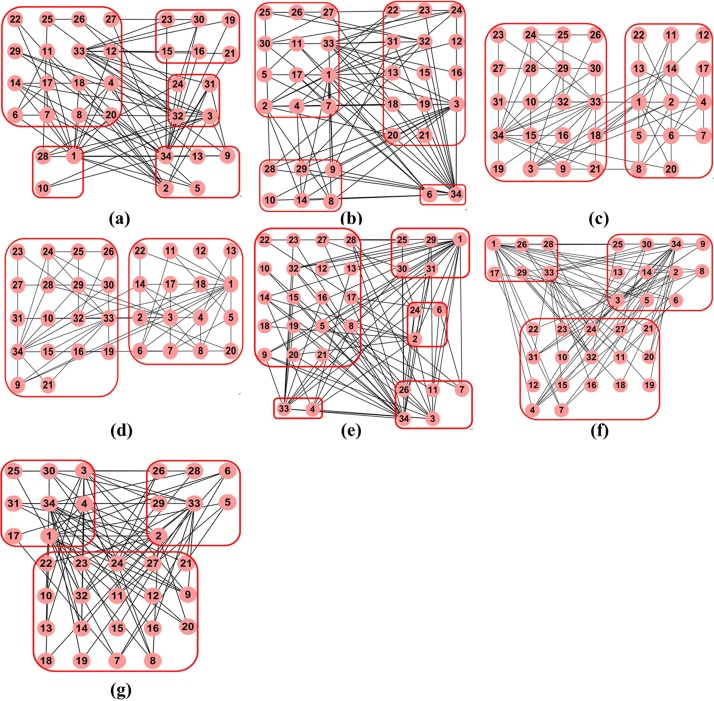
The results of comparison algorithms for *Karate*. **(a)** Spectral. **(b)** Di-Spectral. **(c)** E-Model. **(d)** M-Model. **(e)** LPAD. **(f)** LEOA*. **(g)** LEOA.

To further compare the comparison algorithms, we take assortative network *Karate* as an example and their results are shown in Fig 9. In *Karate*, the disagreement between the administrator (node *v*_1_ and the instructor (node *v*_34_) leads to the division of the network into two groups. We observe that the partitions output by Spectral, Di-Spectral, LPAD, LEOA* and LEOA are anti-community structures, while the partitions output by E-Model and M-Model are community structures. These results indicate that LEOA is capable of exploring anti-community structure in assortative networks. It can be seen that some groups detected by Spectral, Di-Spectral and LPAD consist of two or three nodes, leading to that a few negative relations can be explored in these groups. In addition, we find that only LEOA assigns node *v*_1_ and node *v*_34_ into the same anti-community and reveals the negative relation between the administrator and the instructor. The reason is that node *v*_34_ owns the highest degree (*d*_34_ = 17) in *Karate*. In SCD, node *v*_34_, node *v*_32_ and node *v*_33_ are regarded as structural centers. And then node *v*_1_ is first considered in LAE because it owns the highest degree (*d*_1_ = 16) in the remaining nodes. We find that node *v*_1_ outputs the highest increment of disassortative density when it is added into the group of node *v*_34_ and the group of node *v*_33_. Due to that |*η*_34_|>|*η*_33_|, node *v*_1_ is added into the group of *v*_34_. In GMA, the group memberships of node *v*_1_ and node *v*_34_ are not changed. These results demonstrate that the consideration of node degree in LEOA can help explore the negative relations among objects.

### Efficiency analysis

**Fig 10:**
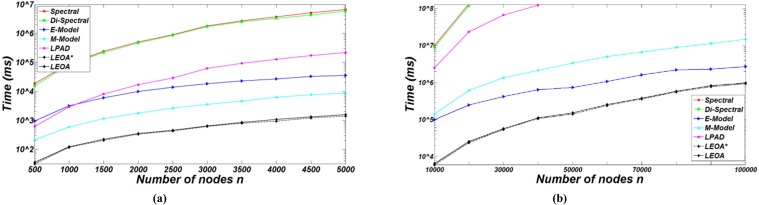
The running time of comparison algorithms on DBM-Net.

In this subsection, we compare the running time of the comparison algorithms on DBM-Net to evaluate the efficiency of LEOA. First, we apply them to DBM-Net with *K* = 2, *m*_*rr*_ = 0, *β* =2, *d*_min_ = 10, *d*_max_ = 50, and *n*∈[500,5000] as shown in Fig 10(A). It can be observed that the running time of E-Model gets close to that of LPAD as *n* increases, but when *n*≥1500, E-Model is more efficient than LPAD. The reason is that LPAD needs *O*(*n*) to determine whether the label of each node is changed in each iteration, so it requires more computational cost than E-Model. In order to validate the performance of comparison algorithms in larger networks, we apply the comparison algorithms to DBM-Net with *n*∈[10000,100000] as shown in Fig 10(B). We find that Spectral and Di-Spectral cannot output the results within 24 hours when *n*≥30000, because with the increase of the number of nodes *n* and the number of edges *m*, the scale of DBM-Net increases and then the running time for calculating the eigenvalues and eigenvectors of the modularity matrix increases greatly. It can be seen that LEOA* requires less running time than LEOA, because the complexity of sorting the nodes in a descending order by the node degree is *O*(*n*log*n*), while the complexity of randomizing the node order for LEOA* is *O*(*n*). From the curves, we can conclude that LEOA is more efficient than five state-of-the-art algorithms in DBM-Net.

## Conclusions

In this paper, we propose a Degree-based Block Model (DBM) for anti-community structure. In DBM, we take the node degree into consideration and obtain a objective function *Q*(*C*) for evaluation. A local expansion optimization algorithm LEOA is designed, in which the nodes with high degree are preferentially considered. Based on the formulation of DBM, a synthetic benchmark DBM-Net is developed for evaluating the algorithms in detecting known anti-community structures. The proposed algorithm LEOA is applied to DBM-Net with up to 100000 nodes and 17 real-world networks and compared with its variant LEOA* and five state-of-the-art anti-community detection algorithms. The experimental results demonstrate the effectiveness and efficiency of LEOA for anti-community detection in networks and exploring negative relations among objects.

There are still some problems to be solved in our future work. First, we find that the edges inside groups have great impacts on the number of structural centers detected by SCD, which leads to the low performance when LEOA is applied to the networks with edges inside groups. In our future work, we plan to employ some priori information by merging some nodes into small groups not to be divided in later operations. This strategy will further improve the effectiveness and efficiency of the algorithm. Second, we find that the number of structural centers detected by SCD is less than the number of anti-communities *K* in the true partitions when *K* is large. In the future, we will divide some groups into two subgroups when the number of edges inside group is more than a certain threshold. Third, it can be seen that the preferential consideration of nodes with high degree can improve the effectiveness of LEOA. However, the node order sorted by the node degree may not output the best result for each network. In the future, we aim to analyze the order of node and select the best node sequence for each network so as to output a better anti-community structure. Finally, DBM-Net is designed based on the assumptions that the group degree and the number of internal edges for each group are the same and each group pair shares the same number of external edges. More complicated benchmark with heterogeneous distribution of group degree and edges number should be considered in the future.

## References

[1] Newman MEJ. The structure and function of complex networks. SIAM Rev. 2003; 45(2): 167–256. doi: 10.1137/S003614450342480

[2] Boccaletti S, Latora V, Moreno Y, Chavez M, Hwang DU. Complex networks: structure and dynamics. Phys Rep. 2006; 424 (4–5): 175–308. doi: 10.1016/j.physrep.2005.10.009

[3] Iyer S, Killingback T, Sundaram B, Wang Z. Attack robustness and centrality of complex networks. PLoS One. 2013; 8(4): e59613 doi: 10.1371/journal.pone.0059613 23565156 10.1371/journal.pone.0059613 PMC3615130

[4] Fortunato S. Community detection in graphs. Phys Rep. 2010; 486(3): 75–174. doi: 10.1016/j.physrep.2009.11.002

[5] Sankowskaa A, Dariusz S. The small world phenomenon and assortative mixing in Polish corporate board and director networks. Physica A. 2016; 443: 309–315. doi: 10.1016/j.physa.2015.09.058

[6] Wu P, Pan L. Multi-objective community detection based on memetic algorithm. PLoS One. 2015; 10(5): e0126845 doi: 10.1371/journal.pone.0126845 25932646 10.1371/journal.pone.0126845 PMC4416909

[7] Newman MEJ. The structure of scientific collaboration networks. Proc Natl Acad Sci U S A. 2001; 98(2): 404–409. doi: 10.1073/pnas.98.2.404 11149952 10.1073/pnas.021544898 PMC14598

[8] Miyauchi A, Kawase Y. Z-score-based modularity for community detection in networks. PLoS One. 2016; 11(1): e0147805 doi: 10.1371/journal.pone.0147805 26808270 10.1371/journal.pone.0147805 PMC4726636

[9] He J, Li C, Ye B, Zhong W. Efficient and accurate greedy search methods for mining functional modules in protein interaction networks. BMC Bioinformatics. 2012; 13 Suppl 10: S19 doi: 10.1186/1471-2105-13-S10-S19 22759424 10.1186/1471-2105-13-S10-S19 PMC3314584

[10] Cunha BR, González-Avella JC, Gonçalves S. Fast fragmentation of networks using module-based attacks. PLoS One. 2015; 10(11): e0142824 doi: 10.1371/journal.pone.0142824 26569610 10.1371/journal.pone.0142824 PMC4646680

[11] Blondel VD, Guillaume JL, Lambiotte R, Lefebvre E. Fast unfolding of communities in large networks. J Stat Mech-Theory Exp. 2008; P10008 doi: 10.1088/1742-5468/2008/10/P10008

[12] Newman MEJ. Finding community structure in networks using the eigenvectors of matrices. Phys Rev E. 2006; 74(3): 036104 doi: 10.1103/PhysRevE.74.036104 17025705 10.1103/PhysRevE.74.036104

[13] Chen L, Yu Q, Chen B. Anti-modularity and anti-community detecting in complex networks. Inf Sci. 2014; 275: 293–313. doi: 10.1016/j.ins.2014.02.040

[14] Zachary WW. An information flow model for conflict and fission in small groups. J Anthropol Res. 1977; 33(4): 452–473.

[15] Trevisan L. Max cut and the smallest eigenvalue. SIAM J Sci Comput. 2012; 41(6): 1769–1786. doi: 10.1137/090773714

[16] Alon N, Sudakov B. Bipartite subgraph and the smallest eigenvalue. Comb Probab Comput. 2000; 9(1): 1–12. doi: 10.1017/S0963548399004071

[17] Holme P, Liljeros F, Edling C, Kim B. Network bipartivity. Phys Rev E. 2003; 68(5): 056107 doi: 10.1103/PhysRevE.68.056107 14682846 10.1103/PhysRevE.68.056107

[18] Wang F. Detecting anti-communities of networks based on spectral method. M.Sc Thesis. Huazhong University of Science and Technology. 2008. Available from: http://cdmd.cnki.com.cn/Article/CDMD-10487-2009227871.htm

[19] Ball B, Karrer B, Newman MEJ. An efficient and principled method for detecting communities in networks. Phys Rev E. 2011; 84: 036103 doi: 10.1103/PhysRevE.84.036103 22060452 10.1103/PhysRevE.84.036103

[20] He D, Liu D, Jin D, Zhang W. A stochastic model for detecting heterogeneous link communities in complex networks. Proceedings of 29th AAAI Conference on Artificial Intelligence. 2015, Jan 25–30; Austin, Texas, USA, pp. 130–136.

[21] Latouche P, Birmele E, Ambroise C. Overlapping stochastic block models with application to the French political blogosphere. Ann Appl Stat. 2011; 5(1): 309–336. doi: 10.1214/10-AOAS382

[22] Karrer B, Newman MEJ. Stochastic blockmodels and community structure in networks. Phys Rev E. 2011; 83(1): 016107 doi: 10.1103/PhysRevE.83.016107 21405744 10.1103/PhysRevE.83.016107

[23] Newman MEJ, Leicht EA. Mixture models and exploratory analysis in networks. Proc Natl Acad Sci U S A. 2007; 104(23): 9564–9569. doi: 10.1073/pnas.0610537104 17525150 10.1073/pnas.0610537104 PMC1887592

[24] Newman MEJ. Communities, modules and large-scale structure in networks. Nat Phys. 2012; 8(1): 25–31. doi: 10.1038/nphys2162

[25] Ren W, Yan G, Liao X, Xiao L. Simple probabilistic algorithm for detecting community structure. Phys Rev E. 2009; 79(3): 036111 doi: 10.1103/PhysRevE.79.036111 19392022 10.1103/PhysRevE.79.036111

[26] Shen H, Cheng X, Guo J. Exploring the structural regularities in networks. Phys Rev E. 2011; 84(5): 056111 doi: 10.1103/PhysRevE.84.056111 22181477 10.1103/PhysRevE.84.056111

[27] Dempster AP, Laird NM, Rubin DB. Maximum likelihood from incomplete data via the EM algorithm. J R Stat Soc Series B. 1977; 39 (1): 1–38.

[28] Goemans MX, Williamson DP. Improved approximation algorithms for maximum cut and satisability problems using semidefinite programming. J Assoc Comput Mach. 1995; 42(6): 1115–1145. doi: 10.1145/227683.227684

[29] Radicchi F, Castellano C, Cecconi F, Loreto V, Parisi D. Defining and identifying communities in networks. Proc Natl Acad Sci U S A. 2004; 101(9): 2658–2663. doi: 10.1073/pnas.0400054101 14981240 10.1073/pnas.0400054101 PMC365677

[30] Newman MEJ. Network data from Newman’s homepage. Available from: http://-personal.umich.edu/~mejn/netdata/, Date of access: 13/04/2017.

[31] Batagelj V, Mrvar A. Pajek datasets. Available from: http://vlado.fmf.uni-lj.si/pub/networks/data/, Date of access: 13/04/2017.

[32] The Koblenz Network Collection. Available from: http://konect.uni-koblenz.de/, Date of access: 13/04/2017.

[33] Danon L, Diaz-Guilera A, Duch J, Arenas A. Comparing community structure identification. J Stat Mech -Theory Exp. 2005; P09008 doi: 10.1088/1742-5468/2005/09/P09008

[34] Albert R, Barabasi AL. Statistical mechanics of complex networks. Rev Mod Phys. 2002; 74(1): 47–97. doi: 10.1103/RevModPhys.74.47

